# Prevalence of mental disorders among older Australians: Contrasting evidence from the 2020–2021 National Study of Mental Health and Wellbeing among men and women and the Health In Men Data Linkage Study

**DOI:** 10.1177/10398562231191692

**Published:** 2023-07-26

**Authors:** Osvaldo P. Almeida, Graeme J. Hankey, Bu B. Yeap, Jonathan Golledge, Christopher Etherton-Beer, Suzanne Robinson, Leon Flicker

**Affiliations:** Medical School, 2720 University of Western Australia, Perth, WA, Australia; and WA Centre for Health & Ageing, 2720 University of Western Australia, Perth, WA, Australia; Medical School, 2720 University of Western Australia, Perth, WA, Australia; and Department of Neurology, Sir Charles Gairdner Hospital, Perth, WA, Australia; Medical School, 2720 University of Western Australia, Perth, WA, Australia; and Department of Endocrinology and Diabetes, Fiona Stanley Hospital, Perth, WA, Australia; Queensland Research Centre for Peripheral Vascular Disease, College of Medicine and Dentistry, 8001 James Cook University, Townsville, QLD, Australia; and Department of Vascular and Endovascular Surgery, The Townsville Hospital, Townsville, QLD, Australia; Medical School, 2720 University of Western Australia, Perth, WA, Australia; and WA Centre for Health & Ageing, 2720 University of Western Australia, Perth, WA, Australia; Deakin Health Economics, 2104 Deakin University, Geelong, VIC, Australia; Medical School, 2720 University of Western Australia, Perth, WA, Australia; and WA Centre for Health & Ageing, 2720 University of Western Australia, Perth, WA, Australia

**Keywords:** depression, anxiety, psychosis, bipolar, alcohol, epidemiology, prevalence, aged

## Abstract

**Objective:**

To determine the prevalence of common mental disorders among older Australians included in the Health In Men Data Linkage Study and compare those with the results of the 2020–2021 National Study of Mental Health and Wellbeing (NSMHW).

**Method:**

We used longitudinal record linkage to estimate the prevalence of mental disorders from age 65 years in a random sample of 38173 Australian men aged 65–85 years living in the Perth metropolitan region. Outcome was the proportion of participants affected by depressive episodes or dysthymia, bipolar disorder, anxiety disorder, psychotic disorder and alcohol use disorder.

**Results:**

Prevalence estimates for participants aged 65–69, 70–74, 75–79, 80–84 and ≥85 years were 0.9%, 2.0%, 3.6%, 5.8% and 12.6% for depressive, 0.2%, 0.3%, 0.4%, 0.4% and 0.7% for bipolar, 0.1%, 0.5%, 1.3%, 2.2%, 6.9% for anxiety, 0.2%, 0.4%, 0.5%, 0.4% and 0.6% for psychotic and 1.2%, 1.7%, 2.1%, 2.2% and 4.2% for alcohol use disorders.

**Conclusions:**

In contrast to the NSMHW, our data indicate that the prevalence of depressive and anxiety disorders increases with age, particularly among the older old. We conclude that the NSMHW should not be relied upon to guide planning or policies to address the mental health needs of older Australians.

The Australian Bureau of Statistics (ABS) has released the results of the 2020–2021 National Study of Mental Health and Wellbeing (NSMHW).^1^ Of the 19.6 million Australians aged 16–85 years, 8.6 million (43.7%) experienced a mental disorder in their life and 4.2 million (21.4%) had relevant symptoms in the 12 months prior to the survey.^1^ The 12-months prevalence varied with age, and was higher among younger than older people (Figure 1).^1^

**Figure 1.**
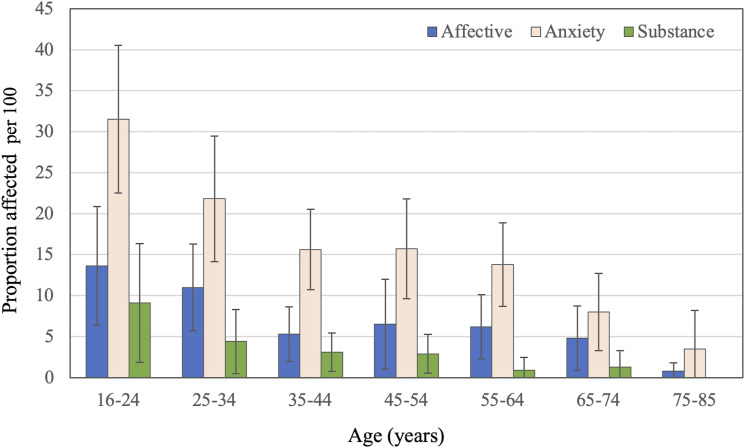
Proportion (bars) of men and women (combined) affected by affective, anxiety and substance use disorder according to the 2020–2021 National Study of Mental Health and Wellbeing. The whiskers represent the 95% confidence interval of the proportion. The data were retrieved from Tables 3.3 and 3.4 of the report.^1^

The NSMHW findings will be used to guide policies and the organisation of mental health services in Australia, but they may have failed to capture the true mental health morbidity of older people.^2,3^ The survey examined 5554 participants, a response fraction of 57.1% of all random household invitations.^4^ This may have introduced healthy participant bias, which is concerning when investigating mental disorders in later life.^5^ The age of participants ranged from 16 to 85 years, and the face-to-face semi-structured and structured assessments were limited to those living independently in the community (1 person per household). Weights were assigned according to the probability of being selected for the study (i.e. if the probability was 1 in 45, the weight of 45 would indicate that the participant represented 45 people living in the community),^4^ and this may have led to increasing bias with increasing age (i.e. healthy older people living in the community would potentially represent a large number of unhealthy older people living in supported accommodation).

Administrative health morbidity data offers an alternative approach to estimating the prevalence of health conditions in later life.^6^ The use of health services increases with age,^7^ so that the opportunity to identify a mental health diagnosis in later life is high. We used the Western Australian Data Linkage System (WADLS) to determine the prevalence of depressive, anxiety, bipolar, psychotic and alcohol use disorders in a cohort of older Australian men aged 65–85 years at recruitment.^8^

## Methods

### Study design, setting and participants

Using the electoral roll, we randomly selected 38,173 Australian men aged 65–85 years living in the Perth metropolitan region in 1996. Participation was limited to men because recruitment sought to investigate the presence of abdominal aortic aneurysm, which is rare among women.^8^ Follow up occurred until death or the 31^st^ of December 2018.

## Outcomes of interest

WADLS includes hospital morbidity data, emergency department and mental health contacts, cancer and death registries.^9^ Coding followed International Classification of Diseases (ICD) guidelines, ICD-9 from 1 January 1970 to 30 June 1999, and ICD-10 from the 1 July 1999. Specific codes identified depressive, bipolar, anxiety, psychotic and alcohol use disorders (Table 1 for details). The same individual could have more than one diagnosis recorded (e.g. alcohol use disorder and depressive disorder). WADLS does not capture primary care data.

**Table 1.** Diagnostic labels of relevant ICD codes

	Code	Diagnosis
Depressive disorder	296.1	Manic-depressive psychosis, depressed type
	298.0	Depressive type psychosis
	300.4	Neurotic depression
	311	Depressive disorders, not elsewhere classified
	F32	Depressive episode
	F33	Recurrent depressive disorder
	F34.1	Dysthymia
Bipolar disorder	296.0	Manic-depressive psychosis, manic type
	296.2	Manic-depressive psychosis, circular type/manic
	296.3	Manic-depressive psychosis, circular type/depressed
	296.4	Manic-depressive psychosis, circular type/mixed
	296.5	Manic-depressive psychosis, circular type/not specified
	F30	Manic episode
	F31	Bipolar affective disorder
Anxiety disorder	300.0	Anxiety states
	300.2	Phobic state
	300.3	Obsessive-compulsive disorders
	F40	Phobic anxiety disorders
	F41	Other anxiety disorders
	F42	Obsessive-compulsive disorder
Psychotic disorder	295	Schizophrenic psychoses
	297	Paranoid states
	298.3	Acute paranoid reaction
	298.9	Unspecified psychosis
	F20	Schizophrenia
	F22	Persistent delusional disorders
	F23	Acute and transient psychotic disorders
	F24	Induced delusional disorders
	F25	Schizoaffective disorders
	F28	Other nonorganic psychotic disorders
	F29	Unspecified nonorganic psychosis
Alcohol use disorder	291	Alcoholic psychoses
	303	Alcohol dependence syndrome
	305.0	Alcohol abuse
	F10	Mental and behavioural disorders due to use of alcohol

**Table 2.** Proportion (%) and 95% confidence interval of the proportion (between brackets) of older men diagnosed with a mental disorder according to their age

	65–69 years	70–74 years	75–79 years	80–84 years	≥85 years
Depression	0.9 (0.9–1.1)	2.0 (2.0–2.3)	3.6 (3.5–3.9)	5.8 (5.5–6.1)	12.6 (11.9–13.3)
Bipolar	0.2 (0.2–0.3)	0.3 (0.2–0.3)	0.4 (0.3–0.4)	0.4 (0.3–0.5)	0.7 (0.5–0.9)
Anxiety	0.1 (0.1–0.1)	0.6 (0.5–0.7)	1.3 (1.2–1.4)	2.2 (2.0–2.4)	6.9 (6.3–7.4)
Psychosis	0.2 (0.1–0.2)	0.4 (0.2–0.3)	0.5 (0.4–0.6)	0.4 (0.6–0.8)	0.6 (1.2–1.8)
Alcohol	1.2 (1.1–1.3)	1.7 (1.6–1.9)	2.1 (1.9–2.2)	2.2 (2.0–2.4)	4.2 (3.8–4.7)
Any disorder	2.3 (2.2–2.5)	4.3 (4.1–4.5)	6.7 (6.4–7.0)	9.4 (9.0–9.8)	20.4 (19.6–21.2)

### Other study measures

We retrieved the dates of birth, contact and death. We used this information to calculate the age of participants at study entry, age at the time of contact with health services and age at the time of death.

### Statistical analyses

We used Stata 17.0 to manage and analyse the data. We examined the proportion of participants with mental health diagnoses across age groups: 65–69, 70–74, 75–79, 80–84 and ≥85 years. Participants contributed mental health data for each of these 5-year periods until death or the 31^st^ of December 2018. The proportion of participants with a recorded diagnosis of depressive, bipolar, anxiety, psychotic and alcohol use disorders were calculated for each age stratum, along with 95% confidence intervals (95%CI) as described by Dean and Pagano.^10^

## Results

**Figure 2.**
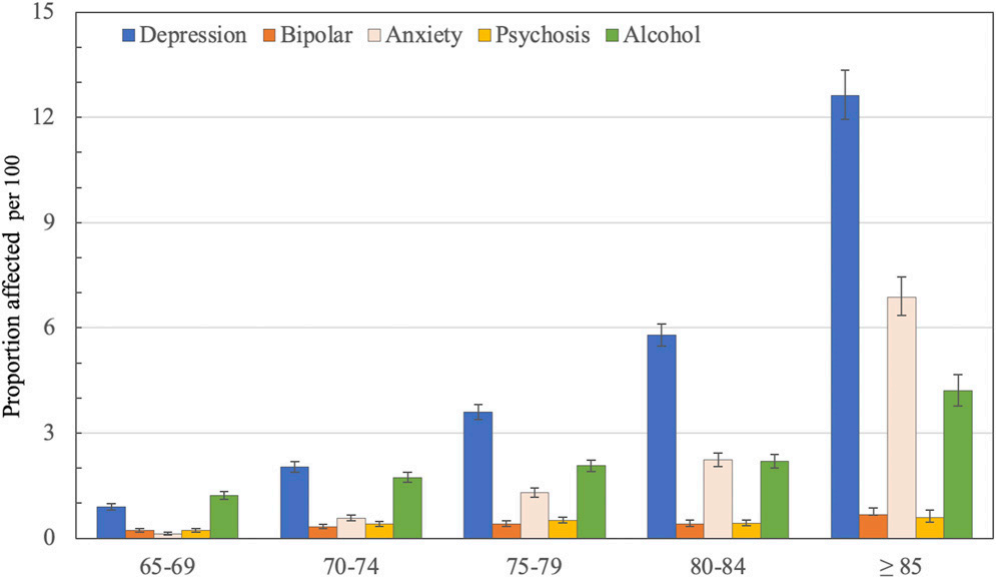
The figure depicts the proportion of participants with a recorded diagnosis of mental disorder according to their age group. The bars indicate the proportion and the whiskers the 95% confidence interval of the proportion (95%CI).

The study included 38173 men (mean age ± SD = 72.5 ± 4.6 years) recruited between the 2^nd^ of April 1996 and the 18^th^ of November 1998. By the end of the follow up, 30410 (79.7%) men had died. The mean age of participants at death was 83.6±6.4 years. During follow up, 35095 (91.9%) men reached age 75 years, 29488 (77.2%) age 80 years, 20966 (54.9%) age 85 years and 7763 (20.3%) survived beyond the age of 85 years Figure 2 depicts the proportion of participants with a recorded clinical diagnosis of depressive, bipolar, anxiety, psychotic and alcohol use disorders according to their age at the time of diagnosis. The proportion of men affected by any of these disorders was 2.3% (95%CI = 2.2–2.5) at age 65–69 years, 4.3% (95%CI = 4.1–4.5) at 70–74, 6.7% (95%CI = 6.4–7.0) at 75–79, 9.4% (95%CI = 9.0–9.8) at 80–84 and 20.4% (95%CI = 19.6–21.2) at age ≥85 years (Table 2).

## Discussion

We found that the prevalence of depressive and anxiety disorders among older men increases sharply after 65 years of age. The prevalence^4^ of bipolar, psychotic and alcohol use disorder remains stable until the early 80 s, but the prevalence of alcohol use disorder increases after the age of 85 years. These findings contrast sharply with those reported by the NSMHW.^1^

While our sample was drawn from the electoral roll, it is subject to selection bias as it did not include women, it was confined to the Perth metropolitan region, ascertainment of diagnoses was between 1996 and 2018, and the diagnosis relied on coding of hospital administrative data (WADLS) according to ICD guidelines. The non-inclusion of women merits discussion. The proportion affected by affective and anxiety disorders in the NSMHW was higher among women than men across all ages, but the pattern of decline with increasing age was the same for both sexes.^1^ Conversely, the proportion of NSMHW men with a recorded 12-months diagnosis of substance use disorder was higher than for women, and was reportedly zero for both in the 75–85 age group.^1^ In contrast, the estimated prevalence of affective, anxiety and substance use disorders among elderly men and women living in Europe was 8.0%, 11.4% and 4.6%.^11^ Evidence from other sources indicates that depression and anxiety are more frequent among older women than men,^12^ suggesting that our results may have underestimated the true size of the mental health problems affecting older Australians.

We acknowledge that depressive and anxiety disorders are commonly managed by general practitioners. Australian general practice activity data for 2015–2016 confirmed that depression and anxiety are among the most frequent reasons for health contacts in primary care, and showed that the frequency of mental health encounters increases with age.^13^ Hence, poor sensitivity of WADLS to detect common mental health diagnoses cannot adequately explain the discrepant findings between the NSMHW and our study (i.e. if WADLS has limited sensitivity, then the true prevalence of mental disorders in older age should be higher than our results suggest). In addition, older adults with mental disorders, such as depression, die earlier than their counterparts.^14^ This could lead to healthy participant bias with increasing age, which suggests that our comparatively high prevalence estimates are not a consequence of differential mortality. Furthermore, while NSMHW participants provided information about the 12-months prevalence of mental disorders, mental disorders recorded by WADLS could range from current to up to 5 years (depending on the age of participants when they joined the study). Hence, the window of opportunity for a mental health diagnosis to be recorded by WADLS would have been greater than for the NSMHW. However, the magnitude of the observed differences seems excessively large to explain away our findings, which are consistent with those of other epidemiological surveys.^13,15–17^

Another issue to consider is the working definition of mental disorder in the NSMHW and the present study. The NSMHW included depressive episode, dysthymia and bipolar disorder under the umbrella ‘affective disorders’, whereas we separated depressive from bipolar disorder, and our definition of depressive disorders included the ICD-9 diagnosis of ‘neurotic depression’ in addition to ICD-10 diagnoses of depressive episode, recurrent depressive disorder and dysthymia. However, only 1 participant was ascribed the diagnosis of ‘neurotic depression’, so that the differences between the studies cannot be explained by a more inclusive definition of depression in our cohort. For anxiety disorders, the NSMHW included panic, agoraphobia, generalised anxiety, obsessive-compulsive and post-traumatic stress disorders, whereas our study excluded from this group participants with stress-related disorders (i.e. the present study was less inclusive that the NSMHW). Likewise, we limited our investigation of the use of substances to disorders associated with alcohol, whereas the NSMHW also included other drug use disorders. Hence, it seems improbable that the higher prevalence of mental disorders observed in our study would be due to a more inclusive approach to the definition of these conditions. The NSMHW’s use of structured clinical interviews for the assessment of mental disorders may have led to a more stringent definition of ‘caseness’, and that use of ICD-9 and ICD-10 codes may have introduced diagnostic inconsistencies that hinder comparisons between the studies. However, this does not seem to provide a sufficiently satisfactory explanation for the differences between the NSMHW and our study. While questions about the validity of mental health diagnoses derived from data linkage may arise, international and Australian data indicate that these datasets generate accurate mental health diagnoses.^18,19^

As frailty is strongly associated with age, admission to residential care and with depression,^20^ it could contribute to the rise in the prevalence of both depression and anxiety after the age of 85 years (depression and anxiety are often comorbid in later life).^21^ This, along the growing prevalence of behavioural and psychological symptoms of dementia with increasing age,^22^ may explain the sharp increase in the prevalence of depressive and anxiety disorders in very late life. The relatively high prevalence of alcohol use disorder in later life, particularly among men, seems to be another health area in need of attention.^17^ Recently released data from the Australian Institute of Health and Welfare showed that 12% of adults older than 70 years consume alcohol at harmful or hazardous levels, and that risky use has been trending upwards over the past 10 years among the old-old.^23^ Finally, we acknowledge that recruitment was limited to metropolitan Perth. While this may raise questions about generalisability, it should be noted that the prevalence of mental disorders across metropolitan regions of Australia is comparable to that of regional and rural areas.^24^

## Conclusion

The NSMHW has generated information that highlights the needs of younger adults, but methodological limitations may have led to substantial underestimation of the prevalence of common mental disorders among older Australians. The evidence from this and other studies indicates that the prevalence of such conditions in later life is high, particularly among the oldest old. Importantly, in this population of older Australian men, the prevalence of common mental disorders rises with age. Our results indicate that the NSMHW should not be relied upon to guide planning or policies designed to address the mental health needs of older Australians.

## Data Availability

The data reported in this study is available to other researchers upon request to Prof. Leon Flicker (leon.flicker@uwa.edu.au).^25^
